# Outcomes of Uretero-ileal Anastomosis in Bladder Cancer Cystectomies: Bricker vs. Wallace 1

**DOI:** 10.7759/cureus.22782

**Published:** 2022-03-02

**Authors:** Siddique Adnan, Muhammad Abu Bakar, Muhammad Arshad Irshad Khalil, Shaukat Fiaz, Zubair Ahmad Cheema, Azfar Ali, Khurram Mir

**Affiliations:** 1 Surgical Oncology, Shaukat Khanum Memorial Cancer Hospital and Research Centre, Lahore, PAK; 2 Urology, Instutute of Kidney Diseases, Hayat Abad Medical Complex, Peshawar, PAK; 3 Biostatistics and Epidemiology, Shaukat Khanum Memorial Cancer Hospital and Research Centre, Lahore, PAK; 4 Urology and Kidney Transplantation, Lahore General Hospital, Ameer Ud Din Medical College, Postgraduate Medical Institute, Lahore, PAK

**Keywords:** bricker, wallace i, bladder cancer, anastomosis, uretero-ileal

## Abstract

Background

The two commonly used methods for uretero-ileal anastomosis (UIA) during radical cystectomy for muscle-invasive bladder cancer (MIBC) are the Bricker and Wallace 1 techniques. Published data on the incidence of strictures at anastomotic sites is limited. This study compares both anastomotic techniques in terms of uretero-ileal stricture (UIS) rates and the factors that govern it in the patient group.

Material and methods

Records of all patients presenting with bladder cancer who underwent radical cystectomy at the department of uro-oncology, Shaukat Khanum Memorial Cancer Hospital and Research Centre (SKMCH&RC) Lahore, Pakistan, from January 1, 2009, to December 31, 2018, were reviewed retrospectively, and all adult patients aged >18 years out of them were selected for the study.

Results

With a total of 116 patients, the mean age was 54.37 ± 11.16 and a male majority (83.6%). Urinary diversion using ileal conduit was performed in 70 (60.3%) patients and the rest of them i.e. 46 (39.7%) had neobladder formation. Amongst them, uretero-ileal anastomosis was constructed via Bricker and Wallace 1 in 73 (62.9%) patients and 43 (37.1%) patients respectively. Pelvic radiotherapy was received by 13 (11.2%) patients. Anastomotic stricture developed in 19 (16.4%) cases. A relatively similar proportion of stricture rate was found in Bricker and Wallace 1 technique (10% vs 13%). Body mass index (BMI) was found to be significantly higher in patients who developed UIS. Incidence of stricture formation was more on the left than right side i.e. 12 (63.2%) vs five (26.3%) while two (10.5%) patients developed bilateral strictures.

Conclusion

No significant difference in stricture formation was noted between Bricker and Wallace 1 technique. High BMI and anastomotic leaks were the contributory factors for this complication during our experience.

## Introduction

Radical cystectomy is the accepted standard of care in patients with muscle-invasive bladder cancer (MIBC) [1]. Presently, orthotropic neobladder and ileal conduit are the two commonly practiced types of urinary diversions in patients undergoing this procedure [2], and the popular methods for performing UIA are the Bricker and Wallace 1 technique [3]. Both techniques are blamed for their potential complications, including the risk of stricture formation, stone, or tumor recurrence [4]. It is therefore essential to know the contributing factors towards them to minimize their occurrence. Studies comparing these techniques are limited [5], and the choice for their selection is based mainly on the surgeons’ experience and preference [6]. Being a referral cancer center, we have reviewed 10 years of our expertise to evaluate the two techniques of anastomoses in uretero-ileal anastomosis (UIS) formation and have assessed the possible contributory factors towards this complication.

## Materials and methods

All patients with MIBC who underwent radical cystectomy from January 2009 through December 2018 at Shaukat Khanum Memorial Cancer Hospital and Research Centre (SKMCH&RC) Lahore, Pakistan, were retrospectively studied after approval from the SKMCH&RC Lahore institutional review board under the approval number (EX-31-12-18-01). Clinical information of patients was collected from the hospital information system. Adult patients with a histologically proven MIBC and a minimum follow-up of six months were selected for the study.

Ileal conduit and orthotopic neobladder urinary diversion and UIA (Bricker and Wallace 1) were performed. The feeding tube of 6 fr was placed across anastomoses and was removed after two weeks postoperatively. All patients were followed up with an ultrasound every three months for UIS evidence in the first year and every six months after that. Patients suspected of UIS were offered percutaneous nephrostomy and antegrade nephrostogram for diagnosis. Imaging by CT scan and MRI scans were performed to rule out tumor recurrence at anastomotic sites. Furthermore, three surgeons performed the surgeries.

The IBM SPSS version 20 (IBM Corp., Armonk, NY, USA) was utilized for statistical analyses. Mean ± standard deviation was employed to summarize quantitative data, whereas frequencies and percentages were used to organize qualitative data. The association of explanatory variables in relation to status (true negative and false negative) was determined by using the chi-square test or Fisher exact test. An independent t-test was used to calculate the mean difference and a p-value of 0.05 was considered statistically significant.

## Results

A total of 116 patients underwent radical cystectomy and urinary diversion with the baseline characteristics described in Table 1.

**Table 1:** Baseline patient’s characteristics N* (number of patients), % (proportion)

Variables	Categories		Total = N* (%)
Age (years)		Mean ± SD	54.37 ± 11.16
		Up to 60 years	71 (61.2%)
		Above 60 years	45 (38.8%)
Sex		Male	97 (83.6%)
		Female	19 (16.4%)
Diversion type		Ileal conduit	70 (60.3%)
		Neo bladder	46 (39.7%)
Anastomosis type		Bricker	73 (62.9%)
		Wallace 1	43 (37.1%)
Body mass index		Mean ± SD	26.15 ± 4.73
Pelvic radiotherapy		No	103 (88.8%)
		Yes	13 (11.2%)
Anastomosis leakage		None	111 (95.7%)
		Left	4 (3.4%)
		Right	1 (0.9%)
Uretero-ileal stricture		Absent	97 (83.6%)
		Present	19 (16.4%)
Overall follow up (months)		Median (range)	48 (24 94)
Time to stricture (months)		Median (range)	12 (5 24)

Table 2 compares variables of patients undergoing UIA by Bricker and Wallace 1 technique. It shows a significant difference in preoperative pelvic radiotherapy in patients undergoing anastomosis by Bricker and Wallace 1 technique, i.e. five (38.5%) and eight (61.5%) respectively. There was no statistically significant difference in UIS formation between the two techniques.

**Table 2:** Comparison of patients characteristics undergoing Bricker or Wallace 1 uretero-ileal anastomosis

Variables	Categories	Bricker = 73 (62.9%)	Wallace 1 = 43 (37.1%)	p-value
Age (years)				0.47
	Mean ± SD	54 ± 11	55 ± 11	
Body mass index				0.63
	Mean ± SD	26.31 ± 4.66	25.86 ± 4.89	
Sex				0.12
	Male	64 (66.0%)	33 (34.0%)	
	Female	9 (47.4%)	10 (52.6%)	
Diversion type				0.02
	Ileal conduit	27 (46.9%)	43 (53.1%)	
	Neo bladder	46 (100.0%)	0 (0.0%)	
Pelvic Radiotherapy				0.03
	None	68 (66%)	35 (34%)	
	Radical	5 (38.5%)	8 (61.5%)	
Stricture				0.62
	Absent	62 (63.9%)	35 (36.1%)	
	Present	11 (57.9%)	8 (42.1%)	

Table 3 describes the relationship between various variables and stricture formation incidence. As shown, BMI and anastomotic leakage were the two factors having a strong association with this complication. Others, i.e. age, gender, diversion type, preoperative pelvic radiotherapy, were not found significant. Left-sided UIS was a more common finding as compared to the right side.

**Table 3:** Comparison of patients with or without stricture undergoing uretero-ileal anastomosis

Variables	Categories	Absent 97 (83.6%)	Present 19 (16.4%)	p-value
Age				0.12
	Mean ± SD	55 ± 11	51 ± 10	
Body mass index				0.001
	Mean ± SD	25.49 ± 4.40	29.46 ± 5.20	
Sex				0.73
	Male	82 (84.5%)	15 (78.9%)	
	Female	15 (15.5%)	4 (21.1%)	
Diversion type				0.78
	Ileal conduit	58 (82.8%)	12 (17.2%)	
	Neo bladder	39 (84.8%)	7 (15.2%)	
Pelvic radiotherapy				0.20
	No	88 (85.4%)	15 (14.6%)	
	Yes	9 (69.2%)	4 (30.8%)	
Stricture site				-
	Left	-	12 (63.2%)	
	Right	-	5 (26.3%)	
	Bilateral	-	2 (10.5%)	
Anastomosis leakage				
	No	96 (86.5%)	15 (13.5%)	0.001
	Yes	1 (20.0%)	4 (80.0%)	

## Discussion

Both Bricker and Wallace techniques were extensively studied since their introduction in 1956 and 1966, respectively, but there is little data on head-to-head comparison between these two techniques in terms of outcomes [7] and therefore the preference of the procedure is mostly by surgeon’s choice. Benign UIS is the most common long-term complication that can lead to a decline in renal function, infection, and a requirement for secondary procedures [8]. Although the exact etiology is not clear, they most likely occur secondary to ischemia at the anastomotic site. Its incidence varies widely and ranges from 2-13% [9], making the real estimate difficult. In literature, the incidence of UIS two years after Bricker and Wallace 1 techniques could be as high as 14% and 11.1%, respectively [10].

Anderson et al. noted that stricture rates are often under-reported owing to non-standardized follow-up duration and imaging [11]. The majority of strictures occur within two years of surgery (median time 7-25 months) and as late as 160 months. This emphasized the need for close postoperative monitoring to ensure early recognition and management [12]. Currently, no published guidelines are available for the optimal follow-up period. To reduce the stricture rates, other methods have been tried without significant success. These include ‘Taguchi’ by Lee et al. and non-refluxing anastomosis by Shaaban et al [13-14]. No difference in rates of stricture formation has been noted between Bricker and Wallace techniques [3]. The left ureter is more at risk, probably contributed by extensive mobilization for retro-sigmoid tunneling [4]. 

No significant association has been found with age, gender, type of urinary diversion (Ileal conduit and orthotropic neobladder), and stricture rate [15]. Obesity (BMI >30kg/m2) is a risk factor due to multiple factors. In these patients, creating anastomosis is challenging because of increased pelvic depth and excessive visceral fats. These hamper gaining adequate length of the left ureter. Additionally, direct pressure over anastomosis due to the mesenteric mass effect precludes tissue healing [16]. It might also be delayed by the release of pro-inflammatory mediators from surrounding mesenteric adipose tissue [17].

Knap et al. reported that UIS's risk was significantly higher in patients with a history of pelvic radiotherapy [5]. It can potentially damage the distal ureter and bowel segment's blood supply, increasing UIS risk and lowering the success rate of repair [18]. It dictates the use of a more proximal healthy bowel segment with better blood supply [19]. Our study's radiotherapy rate, i.e., 12.9%, is comparable with published data (10-20%). The UIS rate was found significant in our study in patients with a history of pelvis radiation 21.1%. Urinary leakage has been associated with an increased risk of UIS. Low surgical techniques, ureteral ischemia are intriguing factors for urinary leakage and subsequent stricture formation. The use of ureteral stents in UIA is argumentative but is thought to ensure accurate alignment and provide mechanical support to the anastomosis, thus preventing urine leakage [20]. Our study's notable limitation is its retrospective nature, non-randomization, and selection bias like patient and technique selection by the surgeon, single center. Secondly, the major limitation of the study would be that age, BMI, sex, diversion type, pelvic radiotherapy, and stricture could become confounding factors, but due to the limited number of patients in each cell (reported in Table 2 and Table 3) multivariate analysis could not be performed. 

## Conclusions

While there is no significant difference in stricture formation in patients having uretero ileal reconstruction by either of Bricker or Wallace 1 techniques, high BMI and anastomotic leaks were the factors found to be mainly responsible for this complication in patients who underwent radical cystectomy for bladder cancer.
